# Implant stability of narrow diameter implants in hyperglycemic patients—A 3‐month case–control study

**DOI:** 10.1002/cre2.587

**Published:** 2022-05-16

**Authors:** Daniel Diehl, Marianna Winkler, Hakan Bilhan, Anton Friedmann

**Affiliations:** ^1^ Department of Periodontology, School of Dentistry, Faculty of Health Witten/Herdecke University Witten Germany; ^2^ Institute of Pharmacology and Toxicology, Center for Biomedical Education and Research (ZBAF), Faculty of Health Witten/Herdecke University Witten Germany

**Keywords:** narrow‐diameter implants, osseointegration, resonance frequency analysis, T2DM

## Abstract

**Objectives:**

The aim of this prospective case–control study was to compare the development of implant stability quotients of narrow diameter implants in patients with type 2 diabetes mellitus (T2DM) and healthy individuals within the first 3 months after implant insertion.

**Methods:**

Sixteen patients with T2DM (HbA1C > 6.5%) as test group and 16 nondiabetic patients (HbA1C < 5.9%) as the control group were evaluated. All patients received narrow‐diameter tissue level implants in an edentulous area posterior to the canine. The implant stability was measured by means of resonance frequency analysis after 3 days, 7 days, 4 weeks, and 3 months postplacement. Statistical analysis of intergroup differences and correlation to HbA1c values and treated jaw was performed in PRISM 8.

**Results:**

The means for implant stability quotients showed a significant increase between Day 3 and 3‐month assessment in both groups. No significant differences between study groups and no correlation of implant stability to HbA1c were found.

**Conclusion:**

The present study shows encouraging clinical outcomes for narrow‐diameter implants inserted in the posterior zone in patients with uncontrolled T2DM.

## 1 INTRODUCTION

Type 2 diabetes mellitus (T2DM) is a metabolic disorder with an increasing prevalence in both developing and developed countries. It is characterized by hyperglycemic blood serum as a result of either insufficient insulin production, defective insulin receptor function, or both (Zimmet et al., 2016). Subsequently, T2DM patients suffer from impaired wound healing due to defective tissue proliferation, remodeling, and exacerbated inflammation (Baltzis et al., 2014).

The number of patients undergoing restorative dental therapy using dental implants has grown significantly during the last decades (Armas et al., 2013). In some cases, bone resorption or periodontitis results in a diminished horizontal and vertical alveolar ridge dimension, making surgical augmentation procedures before implant insertion necessary (Chiapasco et al., 2009). However, extensive reconstructive surgery of the edentulous ridge is not always a viable treatment option. A recent systematic review identified T2DM‐associated vascular and immunological pathologies as a major risk factor for bone augmentation success (Moy et al., 2000).

Narrow‐diameter implants (NDI) were developed for sites with diminished ridge dimensions, which result from numerous clinical reasons and a plethora of studies indicate their clinical success (Klein et al., 2014). By circumventing the need for invasive augmentation procedures and thus the wound healing burden, NDI present a suitable treatment option reducing the wound healing burden in T2DM patients with a diminished alveolar ridge dimension (Friedmann et al., 2021). Recent meta‐analysis and literature reviews attest that NDI are a feasible hardware choice in the posterior region (Schiegnitz & Al‐Nawas, 2018). Moreover, Ma et al. reported that the use of NDI instead of regular diameter implants with bone augmentation procedures did not exhibit differences in survival rates within the reported period (Ma et al., 2019).

Osseointegration, the direct anchorage of the dental implant to the bone, is the major biological prerequisite for implant success. Clinically, successful osseointegration is measurable by implant stability (Albrektsson & Zarb, 1993; Meredith, 1998). In terms of NDI, a study conducted by Pommer et al. showed that a reduced implant diameter had no influence on primary stability as measured by resonance frequency analysis (RFA) (Pommer et al., 2014). However, the clinical literature suggests a significant correlation between reduced implant diameters, the site of implant placement, and declining primary implant stability (Quesada‐García et al., 2012). To this day, studies on the topic of primary implant stability in T2DM patients are scarce. A prospective clinical study by Oates et al. reported a correlation between impaired implant stability and the amount of glycated hemoglobin (HbA1c); however, this study neither focused on the implant diameter nor on chemically modified implant surfaces (Oates et al., 2009). Hence, the aim of this prospective case–control study was to evaluate the implant stability of NDI in relation to the HbA1c and implant position during the first 3 months of implant integration into the native alveolar bone in T2DM and normoglycemic patients.

## 2 MATERIAL AND METHODS

### 2.1 Study design

A total of 32 participants were recruited among patients seeking dental implant treatment within the Department of Periodontology at Witten/Herdecke University (Table 1). Individuals with T2DM (HbA1C > 6.5%) and healthy, nondiabetic persons (HbA1C < 5.9%) missing one or more teeth posterior to the maxillary or mandibular canine and a deficient alveolar ridge were recruited. In each study group, 16 patients with a mean age of 67 were enrolled and matched for age, gender, and prospective implant localization. The individual amount of HbA1c was determined by the patient's physician before enrollment for the study. The absence of T2DM or prediabetes was also verified by consultation with the prospective participant's physician. Exclusion criteria were untreated periodontitis, insufficient oral hygiene, smoking habits, or patients on medication potentially affecting blood perfusion or bone metabolism. The Ethics committee of the Witten/Herdecke University (108/2012) approved the study protocol, and all participants signed the informed consent form.

**Table 1:** Patient demographics

	All groups	Test	Control	*p*
**Patients (dropouts)**	32 (0)	16 (0)	16	0.08*
**Mean age (range)**	67	70 (53–87)	65 (53–84)	
Sex				
Male (%)	14 (48.3%)	10 (61.5%)	6 (37.5%)	0.29*
Female (%)	15 (51.7%)	6 (38.5%)	10 (62.5%)	
**Mean HbA1C (**±**SD)**	‐	7.34 (±0.73)	5.3 (±0.4)	0.0001*
**Jaw (Study implants)**				
Maxilla	19	8 (5)	11 (7)	0.57**
Mandibula	29	15 (11)	14 (9)	
Bone quality				
D1	0	0	0	>0.99**
D2	19	10	9	
D3	12	5	7	
D4	1	1	0	
**Implant total**	48	23	25	
Implant length (mm)				0.34**
8	9	6 (2)	3 (3)	
10	23	10 (8)	13 (9)	
12	16	7 (6)	9 (4)	
**Implant dropouts**	0	0	0	
**Antibiotics**	1	0	1	

* Student's *t*‐test; *α* = .05.

** Fisher's exact test; *α* = .05.

#### 2.1.1 Sample size considerations

The sample size was calculated with G*Power (Faul et al., 2007). For effect size considerations, we referred to the mean maximum change of implant stability relative to baseline as published elsewhere (Oates et al., 2009). However, for our study, we anticipated less significant differences between study groups, due to the chemical modification of the implant surface and the relatively high HbA1C (<8.1%) reported in the previous study. Thus, the anticipated effect size was set at *d* = 1.148, implying a minimum sample size of *n* = 26 (*α* = .05, 1−*β* err prob = 0.8).

### 2.2 Implant surgery

All participants received reduced diameter TiZr Roxolid tissue level implants (3.3 mm, RN TL, SLActive®; Institut Straumann AG, Basel, CH) varying in length from 8 to 12 mm. No additional surgical steps for extending the bone volume at the site of interest were intended by protocol. Placement of all implants was carried out under local anesthesia (Ultracain DS forte®—Sanofi‐Aventis, Frankfurt, Germany) strictly following the standard transmucosal healing protocol in both groups. All surgeries in the study relevant cohort were performed by an experienced periodontist (A.F.) according to the instructions of the manufacturer in a standardized manner. Each patient qualified for one or two adjacent diameter‐reduced implants to be loaded by either a single crown or a fixed partial denture. In the case of two adjacent implants, the most posterior one served as the study implant. All inserted implants were radiographically controlled using the parallel technique for periapical X‐rays after completed surgery. The post‐op regimen included the patient's instruction to abstain from mechanical plaque control in the treated area for 1 week and to use the Chlorhexidine mouth rinse (Chlorhexamed, 0.2%; GlaxoSmithKline Consumer Healthcare GmbH & Co. KG, Munich, Germany) twice a day instead. The administration of systemic antibiotics was restricted to individual needs, there was no prescribing policy by the protocol; analgesic medication (Ibuprofen 600 mg/3× daily) on demand was recommended. Sutures were removed after 7–10 days.

### 2.3 Resonance frequency assessment

**Figure 1:**
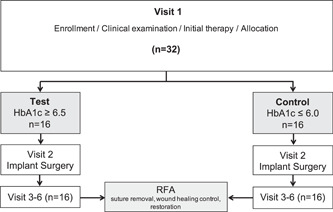
Flow chart of the study protocol. ISQ, Implant Stability Quotient; LDF, Laser Doppler flowmetry; Visit 3, 3 days; Visit 4, 7–10 days; Visit 5, 4 Weeks; Visit 6, 3 months post op.

RFA measurements were performed for each implant 3 days after implant insertion, at Day 7, 4 weeks, and 3 months postplacement (Visit 3–6, Figure 1). For the measurements, the gingiva formers were removed, and magnetic pegs (SmartPeg Type 04; Osstell, Gothenburg, Sweden) were mounted with a special plastic screwdriver. The implant stability quotient (ISQ) of the placed implants was measured (Osstell ISQ meter; Osstell) and recorded according to the manufacturer's instructions. The tip of the instrument was held 1 mm apart from the peg at a 90° angle for a few seconds until the ISQ value was seen on the digital screen. Two measurements per study implant were performed, one at the mesial and one at the buccal aspect. The mean of both values served for further statistical analysis. All measurements were performed by the same, calibrated investigator (M.W.).

### 2.4 Statistical analysis

For all data obtained, mean and standard deviation were calculated. All statistical analyses were performed with GraphPad Prism 8 (GraphPad, San Diego, CA). Statistic methodology included the Shapiro–Wilk, Kolmogorov–Smirnov, and D'Agostino–Pearson tests to evaluate the normality of distribution. For normally distributed data sets, an analysis of variance for repeated measurements followed by Dunnett's multiple comparisons test was performed. The nonparametric Friedman test followed by Dunnett's multiple comparisons test was used to analyze not normally distributed data sets, respectively. Independent variables for RFA data were analyzed using Sidak's multiple comparisons test. Correlations with HbA1c were calculated by Pearson's correlation coefficient. The level of significance was set at *p* = .05.

## 3 RESULTS

Thirty‐two patients with a mean age of 67 years were eligible for further analysis. The mean HbA1c value for the hyperglycemic test group was 7.34% (±0.73). A total of 48 reduced diameter implants were installed and primary stability was achieved. Only one patient in the control group was treated with systemic antibiotics as endocarditis prophylaxis.

**Figure 2:**
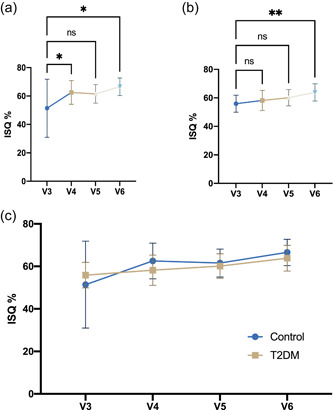
Development of implant stability quotients (ISQ) values in (a) Control, (b) T2DM, and (c) both study groups. Graphs represent means ± standard deviation. **p ≤ .05, *p ≤ .01. T2DM, type 2 diabetes mellitus.

**Figure 3:**
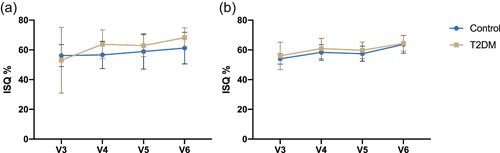
Development of implant stability quotients (ISQ) in (a) mandibular and (b) maxillary implants. Graphs represent means ± standard deviation. **p ≤ .05, *p ≤ .01.

**Figure 4:**
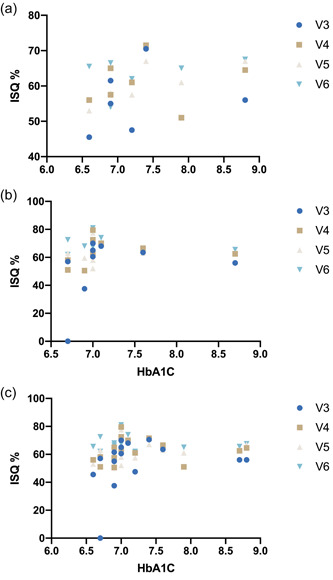
Plotted Pearson correlation matrix. (a) Maxilla, (b) mandibula, (c) both.

The implant stability quotient in both groups increased significantly within the observation period from Day 3 to 3 months (Figure 2). At V3, the groups exhibited mean ISQ values of 51.41 (±20.45) in the control group and 55.87 (±5.99) in the T2DM group (Table 2). According to Dunnet's post hoc test, the control group displayed the most substantial ISQ increase between 3 and 7 days (*p* = .04). The T2DM group exhibited a steadier increase in implant stability, where significant differences were only found between visits 3 and 6 (*p* = .0098). The final resonance frequency assessment before loading the implants by fixed prosthesis yielded an ISQ of 63.84 ± 6.05 in the test group and 66.56 ± 6.18 in the control group. Accordingly, Sidak's multiple comparisons test failed to show significant differences at any time between both groups (Figures 2 and 3). Furthermore, no significant difference in stability was found between implants in the maxilla or the mandible (Figure 3). The Pearson coefficient revealed no significant correlation between HbA1c and ISQ. In the maxilla, however, the implant position was positively correlated with the HbA1c at visit 5 (Figure 4).

**Table 2:** Descriptive statistics of ISQ values in T2DM and control patients.

Group	Visit	Mean ± SD	Mean diff.	*t*	*p* value
T2DM	V3	55.87 ± 5.992	‐		
	V4	58.17 ± 7.090	−2.300	1.049	.3119
	V5	60.13 ± 5.786	−4.258	1.882	.1550
	V6	63.84 ± 6.052	−7.977	3.540	.0098
Control	V3	51.41 ± 9.618	‐		
	V4	58.17 ± 8.374	−2.300	2.992	.0238
	V5	61.53 ± 6.569	−10.13	2.021	.1469
	V6	63.84 ± 6.175	−7.977	3.040	.0216

Abbreviations: ISQ, implant stability quotients; T2DM, type 2 diabetes mellitus.

## 4 DISCUSSION

The objective of this prospective case–control study was to evaluate the osseointegration process of NDI into the native posterior alveolar bone in T2DM and normoglycemic patients. The implant stability quotients were compared between groups based on mean values over a 3‐month observation period and correlated to the underlying HbA1c and the implant‐receiving jaw. The data suggest that NDI display no significant limitations regarding osseointegration quality in T2DM patients. Correspondingly, the data analysis demonstrates that the implant stability quotient is not correlated to the HbA1c amount.

The presented data are not in line with previously published results from a prospective pilot study (Oates et al., 2009). While Oates et al. showed a significantly prolonged implant integration, our analysis yielded no substantial differences. Yet, Oates et al. detected substantial and measurable drawbacks to implant integration only in patients with HbA1c values above 8.1%. In contrast, the mean HbA1c level equaled 7.34% for the hyperglycemic group in our study population. Even though this may serve as an explanation for the conflicting results, it may limit their transferability to patients with a higher level of glycated hemoglobin. Further interpretation of the contradictory outcomes may involve the surface characteristics of the implants used. While Oates et al. used implants with an SLA surface, in this study, we used the hydrophilic SLActive types. At the molecular level, a hydrophilic surface characteristic exerts a proosteogenic and proangiogenic effect on genes relevant for osseointegration. This process is reported to be regulated via PI3K/akt signaling pathways in preosteoblasts (Donos et al., 2011; Gu et al., 2013). Moreover, various preclinical studies affirm the superior properties of the SLActive over the SLA surface in terms of implant integration (Alayan et al., 2017; Schlegel et al., 2013). In light of these findings, the idea that chemically modified surfaces may have ameliorated hyperglycemia‐induced deceleration of peri‐implant bone healing around NDI appears rational. Nevertheless, sufficient randomized controlled clinical trials are lacking to verify this theory indefinitely (Stafford, 2014).

In our study, the mean ISQ value increased constantly in both groups, from 55.87 (±5.992) initially to 63.84 (±6.052) before loading in the T2DM group and 51.41 ± (9.618) to 63.84 ± (6.175) in the control group, respectively (Table 2). In comparison to the values assessed at integrated implants with a greater diameter but similar design, the preload ISQ values in our study were diminished, which may serve as a sign of reduced implant stability (Baldi et al., 2018; Bornstein et al., 2009; Scarano et al., 2006). Nonetheless, the thresholds for appropriate ISQ values obviously differ between various implant systems, and an ISQ range from 55 to 65 is considered safe for Straumann implants according to the published data reviews (Sennerby & Meredith, 2008; Sennerby, 2013). In addition, the implant diameter and insertion torque may also exert a significant influence on the ISQ value (Huang et al., 2020). A recent prospective clinical trial concluded that higher implant diameters are correlated with higher ISQ values (Kim et al., 2017). Therefore, the anticipation of diminished ISQ at NDI appears rational.

Surprisingly, we discovered a significant positive correlation between HbA1c and the ISQ at visit 5 for implants inserted into the maxilla (Figure 4 and Table 3). A previous randomized controlled trial reported a similar observation, disclosing a tendency for higher ISQ values in patients with HbA1c levels exceeding 9.6% compared to patients with HbA1c levels below 9.6% (Khandelwal et al., 2013). However, the authors concluded that varying baseline implant stability quotients may have been the rationale for this finding. In our study, only seven patients received implants in the maxillary area, while neither the patient's age nor the bone quality was taken into account for the calculation. Therefore, the chance that this correlation was detected accidentally is highly probable. Moreover, our finding contradicts the current knowledge and understanding of bone metabolism and biology under diabetic conditions (Hu et al., 2019; Marin et al., 2018). In any case, further research in a larger study population is necessary to substantiate this discovery.

**Table 3:** Pearson correlation values.

Group	Visit	r	95% CI	*p* value
T2DM	V3	0.4180	−0.1364 to 0.7730	.1215
	V4	0.3563	−0.1849 to 0.7317	.1743
	V5	0.4669	−0.05350 to 0.7879	.0696
	V6	0.02151	−0.4916 to 0.5235	.9372
T2DM Maxilla	V3	0.2172	−0.7215 to 0.8746	.6793
	V4	0.1192	−0.6964 to 0.8004	.7991
	V5	0.7984	−0.1138 to 0.9689	.0313*
	V6	0.3531	−0.5448 to 0.8738	.4372
T2DM Mandibula	V3	0.2631	−0.4860 to 0.7893	.494
	V4	0.1446	−0.5747 to 0.737	.7105
	V5	0.05856	−0.6301 to 0.695	.881
	V6	−0.1992	−0.7624 to 0.5358	.6074

* *p* < .05.

Abbreviations: CI, confidence interval; T2DM, type 2 diabetes mellitus.

In this study, all implants were osseointegrated after the 3‐month observation period. In conjunction with the outcome of our analysis, our findings are in line with a variety of original articles. A recent overview of systematic reviews concluded that a hyperglycemic metabolic state had no detrimental effect on the survival rate of dental implants, in spite of the evident vascularization pathology (Souto‐Maior et al., 2019). A variety of preclinical studies support the notion that T2DM patients exhibit impaired wound healing (Komesu et al., 2004; Retzepi et al., 2018). In this regard, the predictability of augmentative procedures in uncontrolled diabetes may be substantially reduced, as adequate wound healing is necessary for graft stability (Mertens et al., 2019). Thus, T2DM patients may benefit from the circumvention of augmentative procedures. Furthermore, numerous studies reported encouraging results for NDI in diabetic patients. In particular, chemical modifications to the implant surface may counterbalance hyperglycemia‐induced impairment of bone healing around dental implants (Cabrera‐Domínguez et al., 2020; Friedmann et al., 2021). Therefore, our study outcome corroborates the previous data: NDI display similar values for the quantitative estimation of osseointegration as measured by ISQ in both, T2DM and healthy patients.

In conclusion, the study demonstrates, that the use of NDI has potential benefits for T2DM patients, as its use may contribute to the reduction of the wound healing burden. However, future clinical trials should focus on the long‐term survival rate of implants functioning under hyperglycemic metabolic conditions.

## AUTHOR CONTRIBUTIONS

The authors are grateful for the contribution of Dr. Kai R. Fischer, who actively took part in performing implant surgeries among the study group population. The narrow diameter implants used in the study are a kind donation from Institut Straumann AG. Open Access funding enabled and organized by Projekt DEAL.

## CONFLICTS OF INTEREST

The authors declare no conflicts of interest.

## ETHICS STATEMENT

This study was performed in line with the Declaration of Helsinki. Approval was granted by the Witten/Herdecke University Ethics Committee (108/2012). Informed consent was obtained from all individual participants involved in the study.

## Data Availability

The data that support the findings of this study are available from the corresponding author upon reasonable request.
